# Medial and lateral knee contact forces during walking, stair ascent and stair descent are more affected by contact locations than tibiofemoral alignment in knee osteoarthritis patients with varus malalignment

**DOI:** 10.3389/fbioe.2023.1254661

**Published:** 2023-09-01

**Authors:** Giordano Valente, Giulia Grenno, Giacomo Dal Fabbro, Stefano Zaffagnini, Fulvia Taddei

**Affiliations:** ^1^ Bioengineering and Computing Laboratory, IRCCS Istituto Ortopedico Rizzoli, Bologna, Italy; ^2^ 2nd Orthopedics and Trauma Unit, IRCCS Istituto Ortopedico Rizzoli, Bologna, Italy

**Keywords:** knee osteoarthritis, knee contact forces, knee contact points, musculoskeletal modeling, stair negotiation, knee force distribution

## Abstract

**Introduction:** Knee OA progression is related to medial knee contact forces, which can be altered by anatomical parameters of tibiofemoral alignment and contact point locations. There is limited and controversial literature on medial-lateral force distribution and the effect of anatomical parameters, especially in motor activities different from walking. We analyzed the effect of tibiofemoral alignment and contact point locations on knee contact forces, and the medial-lateral force distribution in knee OA subjects with varus malalignment during walking, stair ascending and stair descending.

**Methods:** Fifty-one knee OA subjects with varus malalignment underwent weight-bearing radiographs and motion capture during walking, stair ascending and stair descending. We created a set of four musculoskeletal models per subject with increasing level of personalization, and calculated medial and lateral knee contact forces. To analyze the effect of the anatomical parameters, statistically-significant differences in knee contact forces among models were evaluated. Then, to analyze the force distribution, the medial-to-total contact force ratios were calculated from the fully-informed models. In addition, a multiple regression analysis was performed to evaluate correlations between forces and anatomical parameters.

**Results:** The anatomical parameters significantly affected the knee contact forces. However, the contact points decreased medial forces and increased lateral forces and led to more marked variations compared to tibiofemoral alignment, which produced an opposite effect. The forces were less medially-distributed during stair negotiation, with medial-to-total ratios below 50% at force peaks. The anatomical parameters explained 30%–67% of the variability in the knee forces, where the medial contact points were the best predictors of medial contact forces.

**Discussion:** Including personalized locations of contact points is crucial when analyzing knee contact forces in subjects with varus malalignment, and especially the medial contact points have a major effect on the forces rather than tibiofemoral alignment. Remarkably, the medial-lateral force distribution depends on the motor activity, where stair ascending and descending show increased lateral forces that lead to less medially-distributed loads compared to walking.

## 1 Introduction

The onset and progression of knee osteoarthritis (OA) and the consequent degenerative process in the articular cartilage are related, among biological and mechanical risk factors, to the knee contact forces (KCF) of the medial compartment, where the major percentage of the total contact force is transferred (Miyazaki, 2002; Andriacchi and Mündermann, 2006; Felson, 2013). In particular, recent studies showed how medial contact force (MCF) correlates with joint damage and symptom severity (Dell’Isola et al., 2017; Yamagata et al., 2021). Therefore, reducing the MCF has been the focus of several studies to slow down the progression of OA and cartilage damage by using non-invasive strategies such as gait modifications and gait retraining (Fregly et al., 2009; Richards et al., 2018), and joint preserving surgery such as high tibial osteotomy (Whatling et al., 2020; De Pieri et al., 2022).

Musculoskeletal modeling represents a state-of-the-art tool to predict KCF and their distribution in the knee, although the complexity and amount of data required limit its applications (Fregly et al., 2012; Imani Nejad et al., 2020). Therefore, the knee adduction moment has often been used as a surrogate of MCF; however, the level of correlation with MCF is still controversial (Kutzner et al., 2013; Walter et al., 2015), suggesting that the knee adduction moment is not able to explain the variability in the MCF that determines the onset and progression of knee OA, and does not provide any information about the KCF distribution.

KCFs can be altered by joint morphology and limb alignment, which hence represent risk factors for the progression of knee OA (Felson, 2013). The major anatomical parameters of the knee that influence the predictions of medial and lateral KCF include tibiofemoral alignment in the frontal plane (TFA) and contact point (CP) locations, i.e., the centers of pressure between femur and tibia (Gerus et al., 2013; Lerner et al., 2015).

In general, varus TFA was suggested to increase MCF, although the level of correlation found between MCF and TFA is variable. Measurements on patients with total knee replacement showed both marked (Halder et al., 2012) and weak significant correlation between peak MCF and TFA during single-support activities (Kutzner et al., 2017; Trepczynski et al., 2021), and no significant correlation in double-support activities (Kutzner et al., 2017). Perturbation analyses via musculoskeletal modeling showed an increase in peak MCF of 7.7% body-weight (BW)/deg in a total knee replacement patient (Lerner et al., 2015), and 6% BW/deg in both healthy and knee OA patients during walking (Saliba et al., 2017). Varus TFA resulted in significantly increased MCF from 3° upwards in healthy subjects using altered musculoskeletal models (Van Rossom et al., 2019), however a recent modeling study found no significant correlation between KCF and TFA during walking in a cohort of OA patients with a mean 6.3° ± 3.9°varus TFA (Zeighami et al., 2021).

Regarding knee CPs, recent studies showed how CP locations have a significant effect on the prediction of KCF and their distribution. Perturbation analyses showed a decrease in MCF up to 6% BW/mm by shifting the CPs medially while maintaining a constant contact width (Lerner et al., 2015), and up to 4% BW/mm decrease in MCF when increasing medial CPs (Saliba et al., 2017). In addition, significant correlation between peak MCF and CP locations was found in both knee OA and healthy subjects during walking (Zeighami et al., 2021; 2018). CPs are located at the minimum joint space width in the medial and lateral compartments of the knee, and they are typically measured via radiographs, which is the most widespread imaging technique for measuring joint space width and diagnosing OA. In particular, a recent study (Zeighami et al., 2017) calculated knee CP locations of healthy and OA subjects by using bi-planar X-ray images in squat positions, and found medially located CPs in OA subjects, especially on the lateral compartment.

In summary, most studies calculating KCFs and their distribution involve subjects with total knee replacement, there are limited and controversial data on healthy and OA subjects with limited sample sizes, and the few studies analyzing activities different from walking, such as stair ascent and descent, did not include the effect of the anatomical parameters (Meireles et al., 2019; Price et al., 2020). Therefore, it is unclear how variable are KCFs and their distribution when the personalized anatomical parameters are considered during different activities in knee OA subjects, and so is the consequent relationship between KCF distribution and the anatomical parameters.

Therefore, the aim of this study was to analyze the distribution of KCFs during three different motor activities (i.e., walking, stair ascending and stair descending) in a cohort of 51 knee OA patients with varus malalignment, and to evaluate the effects of the anatomical parameters (i.e., TFA and CPs) on the medial and lateral KCFs.

## 2 Methods

### 2.1 Patients and radiographic measurements

**FIGURE 1:**
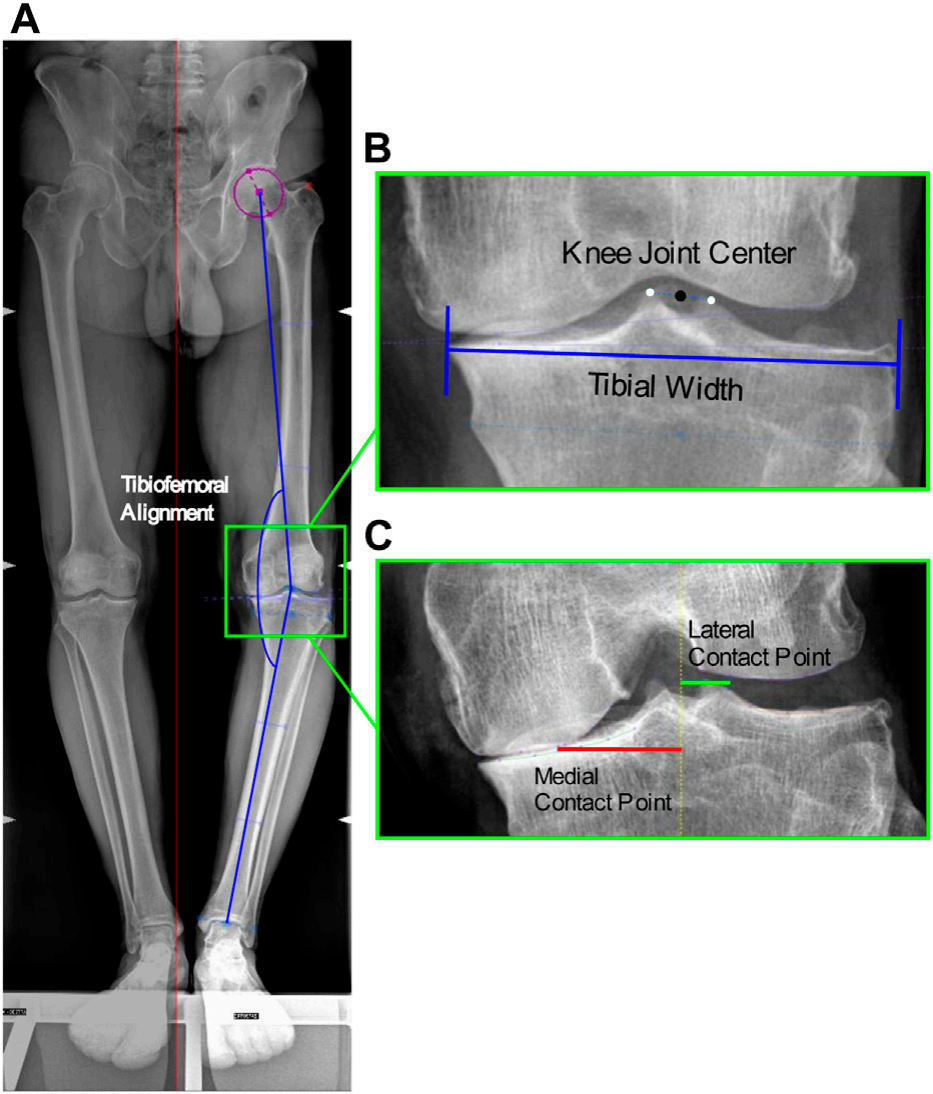
Image-based measurements of the anatomical parameters. **(A)** Tibiofemoral angle from antero-posterior RX: angle between the femoral mechanical axis connecting the hip and knee joint centers, and the tibial mechanical axis connecting the knee and ankle joint centers; **(B)** Knee joint center: midpoint of the centers of the tibial spines, and Tibial width from antero-posterior RX; **(C)** Location of the medial and lateral contact points from Rosenberg RX at the minimum joint space width calculated as the minimum Euclidian distance between the two splines defined on the femoral condyle and tibial plateau, among the interpolated point along the two splines.

A total of 51 patients (42 males, 9 females, mean age 53 ± 8.6 years, mean BMI 26.5 ± 3.9 kg/m^2^) with medial knee OA (graded ≤3 Kellgren-Lawrence), varus malalignment >4°, and no lateral knee OA nor patellofemoral compartment symptoms, participated in this study (Table 1). Long-leg full weight-bearing radiographs and Rosenberg 45° knee flexion radiographs were acquired to measure TFA and CP locations, respectively; in addition, tibial widths were measured on the long-leg radiographs to normalize CP locations (Figure 1).

**TABLE 1:** Characteristics of the 51 knee OA patients with varus malalignment (mean (std)).

				Contact point locations			Contact point locations normalized to tibial width	
Age [yrs]	Gender [F/M]	BMI [kg/m^2^]	Tibiofemoral angle [deg]	Medial [mm]	Lateral [mm]	Tibial width [mm]	Medial [%]	Lateral [%]
53 (8.6)	42 M/9 F	26.5 (3.9)	7.8 (3.5)	34.2 (4.7)	11.7 (1.3)	87.9 (7.6)	39 (5)	13 (2)

TFA was measured as the hip-knee-ankle axis, i.e., the angle between the femoral mechanical axis connecting the hip and knee joint centers, and the tibial mechanical axis connecting the knee and ankle joint centers. The hip center was defined as the center of the circle that best fitted the femoral head; the knee joint center as the midpoint of the centers of the tibial spines; and the ankle center as the mid-width of the tibia and fibula at the level of the plafond (Paley, 2002) (Figure 1). The measured mean TFA was 7.8° ± 3.5°. In addition, the mean tibial widths measured on the radiographs were 87.9 ± 7.6 mm.

Medial and lateral CP locations were identified as the points at the minimum joint space width on the medial and lateral knee compartments. The minimum joint space width was calculated as the minimum Euclidian distance between the two splines defined on the femoral condyle and tibial plateau, among the interpolated point along the two splines (Marsh et al., 2013), implemented in in-house software (Figure 1). The measured mean CP locations medial and lateral of the knee joint center were respectively 34.3 ± 4.7 mm and 11.7 ± 1.3 mm, which corresponded to 39% ± 5% and 13% ± 2% normalized to the tibial widths.

### 2.2 Motion capture data

Motion capture data including 3D marker trajectories, ground reaction forces and EMG activities, were acquired during walking, stair ascent and stair descent for five repetitions each. Walking was performed at self-selected speed; stair ascent and descent was performed step-over-step on a staircase with four steps, each 16 cm high, 28 cm deep and 86 cm wide, with no railings nor banisters, and with two force plates under the second and third step. The patients were first instrumented with 22 reflective markers on pelvis and lower limbs according to the established IORgait marker set and protocol (Leardini et al., 2007). Then motion capture data were simultaneously collected using an 8-camera motion capture system (100 Hz, Vicon 612 Motion System, Oxford, United Kingdom), two embedded force plates (2000 Hz, Kistler, Winterthur, Switzerland), and surface EMG (2000 Hz, Wave Wireless, COMETA, Milan, Italy) through adhesive disposable electrodes placed according to the SENIAM recommendations, from the following muscles: gluteus medius, erector spinae, rectus femoris, vastus medialis, biceps femoris, semitendinosus, medial gastrocnemius and tibialis anterior.

EMG signals were first detrended, band-pass filtered (40–200 Hz), full wave rectified and low-pass filtered at 6 Hz with a 6^th^ order Butterworth filter to obtain envelopes (Winter, 2009). Then, to account for physiological electromechanical delay (Corcos et al., 1992), time-shifting in a range of 10–100 ms was applied to the EMG envelopes corresponding to the highest cross-correlation coefficient of two time sequences (Żuk et al., 2018).

### 2.3 Musculoskeletal modeling and simulations of motor activities

A freely-available and validated full-body musculoskeletal model including 18 body segments and 92 musculotendon actuators (Lerner et al., 2015) was used for this study, in conjunction with experimental 3D marker trajectories and ground reaction forces to ultimately calculate joint reaction forces during the different motor activities, by implementing an optimization-based inverse-dynamics workflow in OpenSim (Delp et al., 2007). The musculoskeletal model includes the possibility to personalize the TFA based on the image measurements, and an augmented mechanism in the tibiofemoral joint model (i.e., additional bodies and joints) to solve for medial and lateral knee contact forces, allowing personalization of medial and lateral CP locations (Lerner et al., 2015).

To evaluate the effect of TFA and CPs, we created a set of musculoskeletal models for each patient and performed simulations with the following four conditions of personalization.

• Uninformed model (UI): This model included the baseline values of 0° TFA and CP locations of 20 mm medial and lateral of the knee joint center (Lerner et al., 2015), with no personalization of the knee anatomical parameters.
• TFA-informed model (TFAI): This model included the personalization of the TFA of both limbs for each patient, according to the radiographic measurements.
• CP-informed model (CPI): This model included the personalization of the medial and lateral CP locations of both limbs for each patient, according to the radiographic measurements.
• Fully-informed model (FI): This model included the full personalization of TFA and CP locations.

First, the models were scaled to each subject by scaling the dimensions of each body segment, mass and inertial properties, and the elements attached to the body segments, based on (i) the distances between the experimental markers from the static trial and the corresponding virtual markers on the model, and (ii) body mass. Joint angles during the motor activities were then calculated through Inverse Kinematics, by minimizing the errors between experimental and virtual markers. Then, muscle forces were calculated by decomposing the joint moments among the musculotendon actuators through Static Optimization, by minimizing the sum of muscle activations squared and accounting for the force–length–velocity relationship (Anderson and Pandy, 2001). Finally, the medial, lateral and total knee contact forces were calculated from the instantaneous force equilibrium through Joint Reaction Analysis (Steele et al., 2012). When the models predicted physiologically impossible tensile LCFs, the LCF was constrained to zero (i.e., unloaded), and a tensile force representing the collateral ligament was recruited to maintain equilibrium (Brandon et al., 2014). In addition, the muscle activations predicted by the models were used in a quantitative comparison with the corresponding processed EMG data to indirectly validate the modeling outputs of KCFs. This indirect validation is presented in the Supplementary Material S1.

### 2.4 Data analysis

First, all KCFs were normalized to the percentage of stance phase of motor activity cycle and to each subject body-weight (BW). To evaluate the effect of TFA and CP on MCF and LCF, the forces from all models were first expressed as mean and standard deviation among the patients, and plotted. Then statistical parametric mapping (Nichols and Holmes, 2002) was used to evaluate statistically significant differences in KCFs among the models across the motor activity cycles. Specifically, non-parametric repeated measures ANOVA and associated *post hoc* analysis, i.e., non-parametric two-tailed paired t-tests, were conducted among the forces obtained from the four models, by using the SPM1D package (SPM, www.spm1d.org, v0.4 (Pataky et al., 2013)) implemented in MATLAB. The differences were considered clinically relevant if significant differences occurred for at least a consecutive 4% of the motor activity cycle (Wesseling et al., 2018; Valente et al., 2021).

To analyze the effect on the distribution of the KCF peaks, the two force peaks corresponding to the typical double-bump force pattern across the motor activity cycles were calculated and presented as boxplot distribution with quartiles. Then Wilcoxon signed-rank tests (α = 0.05) were applied to evaluate statistically significant differences between each pair of models.

In addition, to analyze how KCFs were distributed during the three motor activities, the medial-to-total contact force ratios (MFRatio) were calculated from the FI model outputs across the stance phase of walking, stair ascending and stair descending, and averaged among the patients. In addition, the MFRatio at the two force peaks were calculated and presented as boxplot distribution with quartiles.

Finally, to evaluate the relationship between the anatomical parameters and KCFs, a multiple regression analysis was performed. The independent variables were TFA, normalized medial CP and normalized lateral CP, and the dependent variables were MCF, LCF and MFRatio at the two force peaks from the FI model outputs. The coefficients of determination R^2^ and the coefficient estimates of each independent variable with the corresponding *p*-values were calculated in the linear regression model (i.e., y = c_0_ + c_1_x_1_ + c_2_x_2_ + c_3_x_3_). Variance inflation factors were also calculated to measure multicollinearity among the independent variables in the multiple regression model.

## 3 Results

### 3.1 Effect of the anatomical parameters on KCFs

**FIGURE 2:**
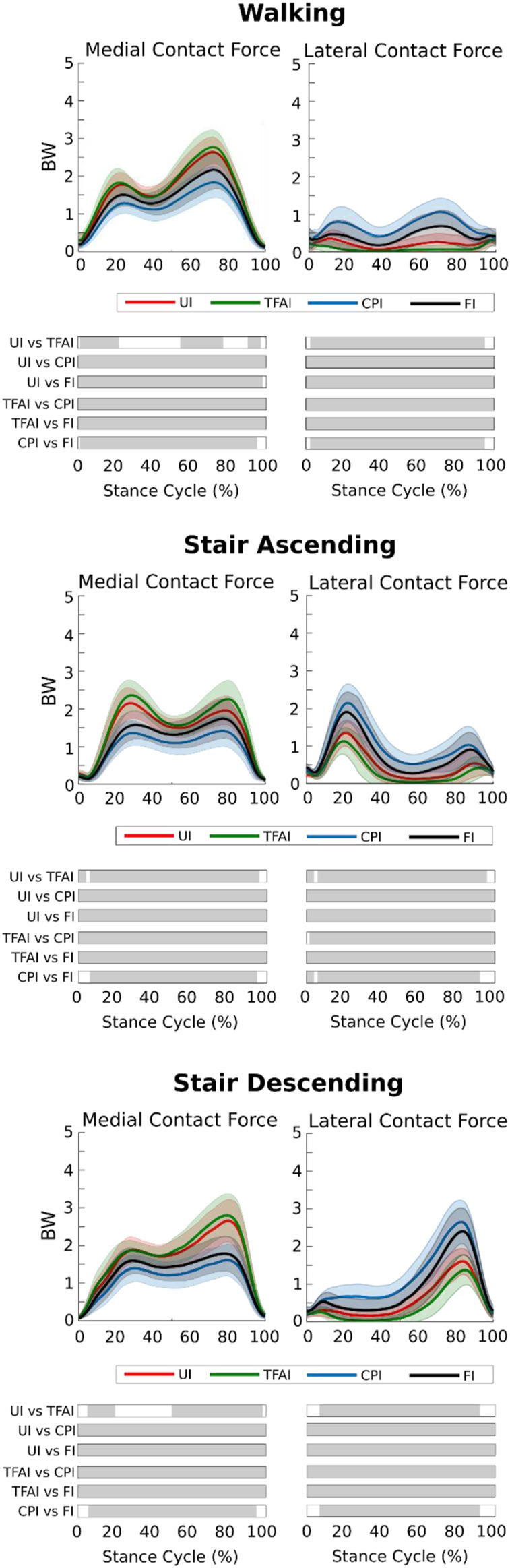
Medial and lateral knee contact forces from the four models with different levels of personalization during the three motor activities (mean ± std). Time intervals during stance with statistically significant differences (*post hoc* SPM t-tests) are reported as Gy bars below each subplot.

**FIGURE 3:**
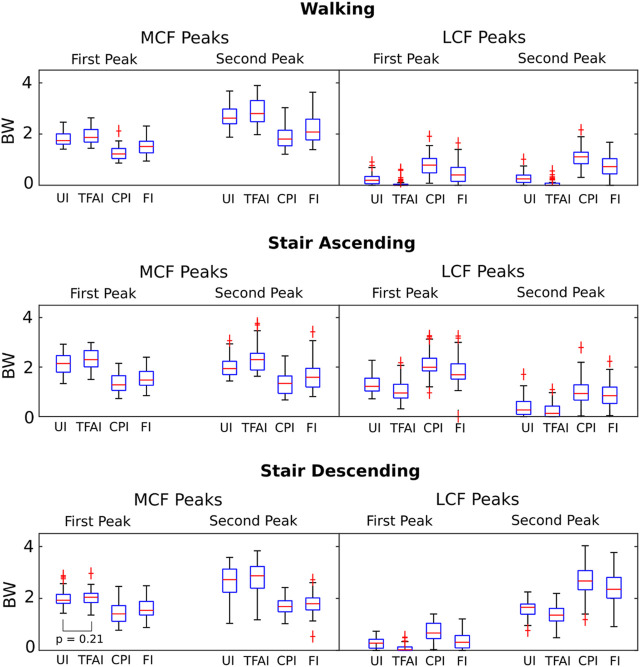
Boxplot distributions of the medial and lateral knee contact forces from the four models with different levels of personalization at the 1^st^ and 2^nd^ force peaks during the three different motor activities. All distributions are significantly different (Wilcoxon signed-rank tests) among the four models, except the one indicated.

We found that TFA and CP locations significantly affected the predicted KCFs during all three motor activities. SPM non-parametric ANOVA among models showed significance for the whole stance phase of the gait cycles, and the *post hoc* comparisons of the forces between models showed significant differences in most of the stance phases in all cases (Figure 2). Focusing on the peaks of KCFs, we found that all distributions were significantly different among the four models, except from the first peak of MCF between UI and TFAI (Figure 3; Table 2). The largest force differences always occurred between TFAI and CPI model outputs, whose medians of KCF distributions reached a difference of 1.2 BW in MCF and 1.3 BW in LCF during stair descending (Figure 3; Table 2).

**TABLE 2:** Medial and lateral knee contact forces from the 4 models with different levels of personalization at the 1^st^ and 2^nd^ force peaks during the three motor activities (Median and Interquartile Range). UI: Uninformed, TFAI: Tibiofemoral-Informed, CPI: Contact-Points-Informed, FI: Fully-Informed models.

Medial contact force					Lateral contact force			
1st peak			2nd peak		1st peak		2nd peak	
Median		IQR	Median	IQR	Median	IQR	Median	IQR
Walking								
UI	1.74	0.40	2.62	0.58	0.20	0.28	0.25	0.29
TFAI	1.87	0.49	2.80	0.83	0.00	0.04	0.00	0.09
CPI	1.22	0.39	1.80	0.61	0.79	0.56	1.11	0.45
FI	1.52	0.45	2.08	0.81	0.41	0.55	0.73	0.59
Stair Ascending								
UI	2.14	0.67	1.94	0.54	1.22	0.52	0.27	0.52
TFAI	2.30	0.66	2.30	0.68	0.95	0.56	0.13	0.42
CPI	1.28	0.59	1.34	0.70	1.99	0.51	0.93	0.62
FI	1.47	0.56	1.59	0.76	1.69	0.62	0.85	0.66
Stair Descending								
UI	1.93	0.35	2.72	0.89	0.28	0.34	1.66	0.39
TFAI	2.05	0.36	2.87	0.84	0.02	0.14	1.36	0.48
CPI	1.40	0.60	1.69	0.42	0.67	0.59	2.67	0.74
FI	1.54	0.53	1.79	0.46	0.31	0.48	2.35	0.80

In general, we found that the introduction of TFA in the models led to a systematic increase in MCF and a decrease in LCF, while, on the contrary, the introduction of CPs systematically decreased MCF and increased LCF. Specifically, the introduction of CPs always led to more marked variations in KCFs compared to the introduction of TFA. Indeed, passing from UI to TFAI always led to less variations in KCFs than passing from UI to CPI, and FI model outputs were always closer to CPI than TFAI model outputs (Figures 2, 3; Table 2).

### 3.2 KCF medial-lateral distribution and relationship with the anatomical parameters

**FIGURE 4:**
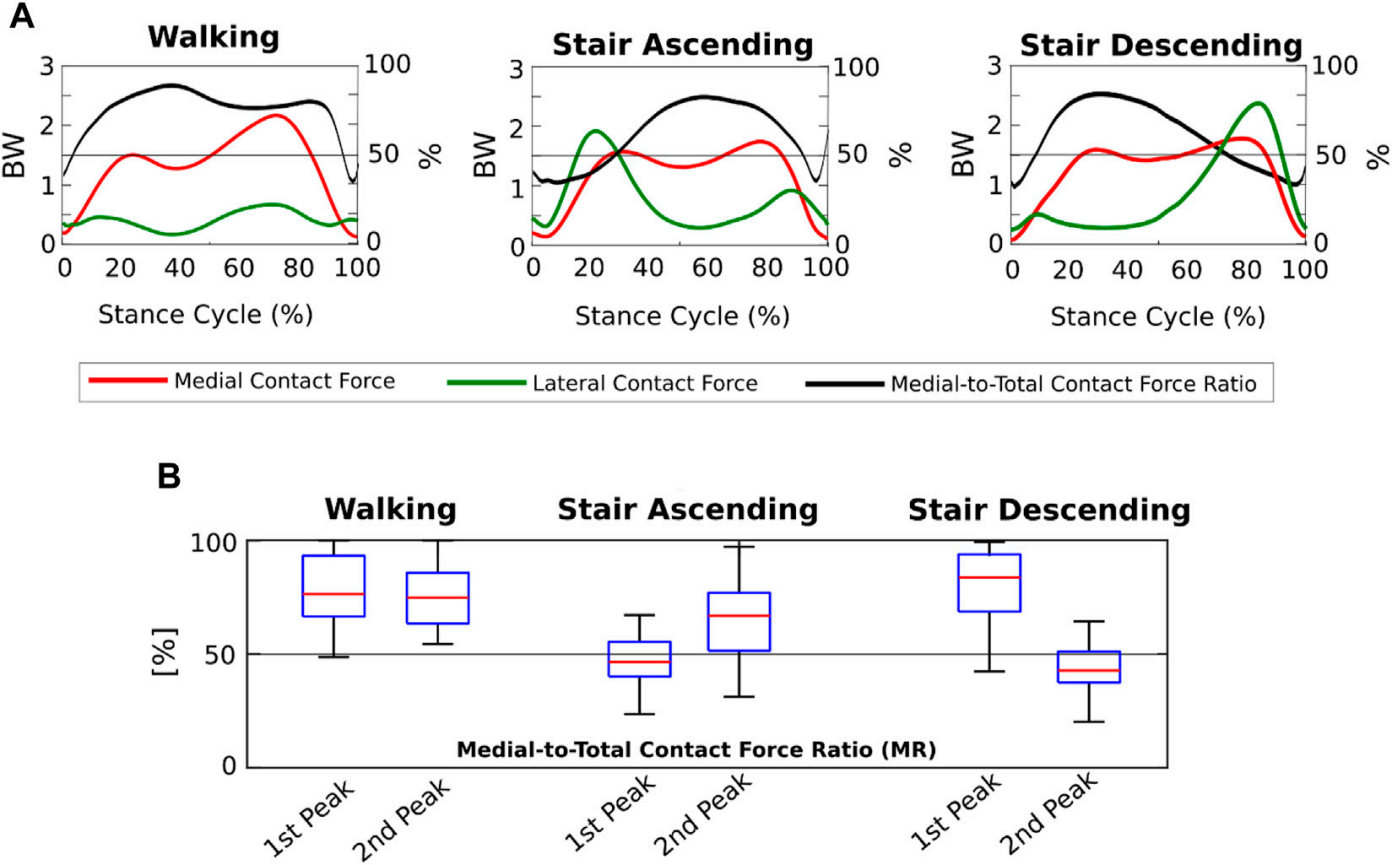
Medial-lateral distribution of the knee contact forces from the Fully-Informed model. **(A)** Medial and lateral contact forces (BW), and medial-to-total contact force ratios (MFRatio) (%) across the three motor activity cycles; **(B)** Boxplot distributions of the medial-to-total contact force ratios (MFRatio) (%) at the 1^st^ and 2^nd^ force peaks during the three different motor activities.

We found that the KCFs had different medial-lateral distributions according to the motor activity analyzed. Indeed, focusing on the FI model outputs, the mean MFRatio was over 50% (i.e., more medially distributed KCFs) for the 90% of the stance phase of walking, which decreased to the 65% of stair ascending and the 64% of stair descending (Figure 4A). Focusing on the KCF peaks, we found a median MFRatio value of 75% (IQR 22%) at the highest force peak during walking, which decreased to 47% (IQR 15%) during stair ascending, and 43% (IQR 14%) during stair descending (Figure 4B).

Regarding the relationship between anatomical parameters and KCFs, we found significant R^2^, ranging from 0.3 to 0.67, at both force peaks during all motor activities in the multiple regression analysis (Table 3). Specifically, for the MCF, we found significant coefficients of the anatomical parameters for the medial CP only, except one case for the TFA, while for the LCF and MFRatio, we found significant coefficients for TFA and medial CP; the coefficients of the lateral CP were not significant except two mild cases for the MFRatio (Table 3). The Variance inflation factors were 1.52 for TFA, 1.49 for medial CP and 1.12 for lateral CP, indicating low collinear relationships among anatomical parameters.

**TABLE 3:** Multiple regression analysis (y = c_0_ + c_1_x_1_ + c_2_x_2_ + c_3_x_3_) including the anatomical parameters as independent variables (X) and the knee forces as dependent variables (y) at the 1^st^ and 2^nd^ force peaks during the three different motor activities. Y-intercepts and regression coefficients for each anatomical parameter with the corresponding *p*-values, and R^2^ with the corresponding *p*-values are reported in the table. Statistically-significant values are highlighted in bold. TFA: Tibiofemoral Angle, nCP med: Normalized Medial Contact Point, nCP lat: Normalized Lateral Contact Point.

Walking												
	Medial contact force				Lateral contact force				Medial-to-total ratio			
	First peak		Second peak		First peak		Second peak		First peak		Second peak	
	Coeff	*p*	Coeff	*p*	Coeff	*p*	Coeff	*p*	Coeff	*p*	Coeff	*p*
Intercept	2.500	**0.000**	4.054	**0.000**	−0.043	**0.000**	0.708	**0.000**	1.091	**0.000**	0.848	**0.000**
TFA	0.016	0.170	0.034	0.087	−0.040	**0.031**	−0.069	**0.000**	0.017	**0.009**	0.022	**0.000**
nCPmed	−3.769	**0.000**	−6.310	**0.000**	2.760	**0.014**	3.228	**0.002**	−1.499	**0.000**	−1.232	**0.000**
nCPLat	2.920	0.180	2.707	0.461	−2.157	0.525	−5.384	0.081	1.156	0.335	1.694	0.048
R^2^	0.55	**0.000**	0.57	**0.000**	0.35	**0.000**	0.57	**0.000**	0.51	**0.000**	0.67	**0.000**

Stair Ascending												
	Medial Contact Force				Lateral Contact Force				Medial-to-Total Ratio			
	First Peak		Second Peak		First Peak		Second Peak		First Peak		Second Peak	
	Coeff	*p*	Coeff	*p*	Coeff	*p*	Coeff	*p*	Coeff	*p*	Coeff	*p*
Intercept	2.535	**0.000**	2.404	**0.000**	2.736	**0.000**	1.168	**0.000**	0.560	**0.000**	0.722	**0.000**
TFA	−0.001	0.956	0.078	**0.001**	−0.063	**0.029**	−0.076	**0.002**	0.009	**0.026**	0.028	**0.000**
nCPmed	−4.072	**0.000**	−4.187	**0.002**	3.211	0.062	2.064	0.146	−1.051	**0.000**	−1.053	**0.010**
nCPLat	4.109	0.174	2.480	0.546	−11.905	0.300	−3.966	0.374	1.757	**0.020**	1.087	0.381
R^2^	0.37	**0.000**	0.55	**0.000**	0.30	**0.002**	0.37	**0.000**	0.54	**0.000**	0.56	**0.000**

Stair Descending												
	Medial Contact Force				Lateral Contact Force				Medial-to-Total Ratio			
	First Peak		Second Peak		First Peak		Second Peak		First Peak		Second Peak	
	Coeff	*p*	Coeff	*p*	Coeff	*p*	Coeff	*p*	Coeff	*p*	Coeff	*p*
Intercept	2.781	**0.000**	3.055	**0.000**	−0.283	**0.000**	2.884	**0.000**	1.227	**0.000**	0.566	**0.000**
TFA	0.018	0.239	−0.014	0.503	−0.016	0.298	−0.080	**0.011**	0.006	0.351	0.008	**0.031**
nCPmed	−4.773	**0.000**	−4.773	**0.000**	3.676	**0.000**	3.952	**0.027**	−1.867	**0.000**	−1.098	**0.000**
nCPLat	4.255	0.147	5.221	0.203	−4.060	0.162	−10.172	0.082	1.667	0.195	1.788	**0.010**
R^2^	0.56	**0.000**	0.30	**0.002**	0.44	**0.000**	0.38	**0.000**	0.49	**0.000**	0.61	**0.000**

## 4 Discussion

In this study, we analyzed the effect of knee anatomical parameters of TFA and CP locations on the medial and lateral knee contact forces, and how these medial and lateral forces were distributed and related to the anatomical parameters during walking, stair ascending and stair descending in a cohort of 51 knee OA patients with varus malalignment. To achieve our objective, we used musculoskeletal modeling with image-based personalization of the anatomical parameters to calculate medial and lateral knee contact forces during the three motor activities.

Overall, we found that medial and lateral knee contact forces are more affected by the location of knee CPs than TFA in knee OA patients with varus malalignment, and medial-lateral force distribution varies with the motor activity performed by the patients, where the major responsible is the location of the medial CPs, especially in more demanding motor activities.

Introducing personalized CP locations in our models led to significantly decreased loads on the medial compartment of the knee and significantly increased loads on the lateral compartment, with an effect that was always markedly larger than the opposite effect of introducing personalized TFA in all motor activities (Figures 2, 3; Table 2). Our study is the first one analyzing the effect of TFA and CP locations on KCFs in patients with varus malalignment not only during walking, i.e., also stair ascending and descending, showing a comparable effect and even more marked of what found during walking. For example, at the highest force peak of stair descending, the MCF showed a decrease of 1 BW passing from the uninformed to the contact-point-informed model and an increase of only 0.1 BW passing from the uninformed to the tibiofemoral-alignment-informed model, while the fully-informed model showed a 0.9 BW decrease from the uninformed model (Figure 3; Table 2). Consequently, including personalized locations of CPs is crucial in the analysis of KCFs of patients with varus malalignment. Previous sensitivity studies that used the same baseline model have shown the same trend on the medial compartment of decreased loading when the medial CPs are more medially located and increased loading when the varus TFA increases (Lerner et al., 2015; Saliba et al., 2017). We found good agreement on the effect of CPs from our knee OA cohort with the reported MCF sensitivity of −0.04 BW/mm to medial CPs and 0.008 BW/mm to lateral CPs, but less agreement on the effect of TFA with the reported MCF sensitivity to TFA of 0.06 BW/deg during walking (Saliba et al., 2017), as we found less force variation related to TFA (i.e. 0.02 BW/deg). However (Saliba et al., 2017), performed a sensitivity analysis including a majority of healthy subjects (14 healthy and 9 knee OA subjects), while our study included a larger cohort of 51 knee OA patients with varus malalignment with smaller TFA dynamics, which could explain this difference.

The medial-lateral distribution of the KCFs passed from markedly medial during walking to slightly medial during stair ascending and descending, where the forces were even more laterally distributed at the force peaks. We found that while during walking the 90% of the gait cycle showed KCFs more medially distributed, during stair ascending and descending a markedly different trend occurred (Figure 4). Indeed, on average the LCFs were larger than the MCFs around the force peaks, i.e., MFRatio below 50%, leading to an overall less medially distributed load across the cycles of stair ascending and descending (Figure 4). Previous research on medial-lateral KCF distribution in OA subjects via musculoskeletal modeling showed mean MFRatio ranging from 63% to 87% on the force peak during walking (Kumar et al., 2013; Sritharan et al., 2017; Van Rossom et al., 2018; Zeighami et al., 2021), which is in agreement with our findings (i.e., MFRatio of 75%). In addition, we found agreement on the different medial-lateral KCF distribution during stair ascending with a previous study showing MFRatio slightly below 50% especially on the force peak in 10 symptomatic OA subjects (Price et al., 2020). Conversely, our findings differed from those of the other study analyzing stair negotiation (Meireles et al., 2019), where the authors found MCFs larger than LCFs on the force peak of stair ascending and descending. However, only 5 patients (with bilateral OA) were included, and without personalization of TFA and CP locations in the models, which could have markedly affected how medial-lateral forces were distributed, especially during stair negotiation activities.

We found that the anatomical parameters explain approximately the 30%–67% of the variability in the knee forces analyzed, as suggested by the R^2^ found in the multiple regression analysis (Table 3). The location of the medial CPs was the anatomical parameter that best predicted medial contact forces, showing significant coefficients at both force peaks during all motor activities. TFA and medial CPs have a comparable effect on lateral contact forces and medial-to-total force ratio, while lateral CPs have a negligible effect on all knee forces (Table 3). In agreement with our findings, a recent study on a smaller cohort of OA subjects during walking showed that the medial CP locations have larger effect than TFA on KCFs, although they found no significant correlation between TFA and KCFs, likely due to the lower TFA variability of their smaller cohort (Zeighami et al., 2021).

The locations of knee CPs that we identified in our patients via radiographic measurements (Table 1) were in good agreement with those found in recent research using biplanar radiographic images in different squat positions and including 9 OA patients (Zeighami et al., 2017). Indeed, the authors found medial and lateral CPs of 36% and 12% normalized to the tibial width medial and lateral of the knee center at 45° knee flexion squat, very close to 39% and 13% of our subjects from the Rosenberg 45° knee flexion position. In fact, although our absolute CP locations were larger than those from the previous study, we found bigger tibial widths in our cohort, likely due to the large majority of males compared to the large majority of females included in (Zeighami et al., 2017).

Our predictions of medial and lateral contact forces have some limitations. First, our knee models did not include CP locations varying with the knee flexion angle. Although a recent study found that CP trajectory can lead to a few significant differences in MCF peaks in healthy subjects (Zeighami et al., 2018), the same authors found no significant differences in medial CPs among different squat positions in OA subjects and a significant difference in lateral CPs between 70° and 0° knee flexion, confirming a good identification of our CPs included in the models. In addition, we identified CPs on 2D radiographic measurements, which were not validated against more accurate 3D measurements, although the above-discussed comparison with CP locations from more accurate measurements (Zeighami et al., 2017) showed very close values.

In conclusion, in this study we found that in knee OA subjects with varus malalignment, the location of knee contact points, especially on the medial compartment, has a major effect on medial and lateral knee contact forces rather than tibiofemoral alignment, and the medial-lateral force distribution depends on the motor activity, where stair ascending and descending show increased lateral forces that lead to less medially-distributed loads compared to walking. Further analyses including the relationship with kinematics and kinetics parameters will help explain this mechanism. We found that including personalized locations of CPs is crucial in the analysis of knee forces in patients with varus malalignment, and evaluating the accuracy of image-based identification of CP locations will consequently help improving the accuracy of force predictions.

## Data Availability

The raw data supporting the conclusion of this article will be made available by the authors, without undue reservation.
