# Supplementary material for: Medial and lateral knee contact forces during walking, stair ascent and stair descent are more affected by contact locations than tibiofemoral alignment in knee osteoarthritis patients with varus malalignment
Source: Front Bioeng Biotechnol. 2023 Sep 1;11:1254661. doi: 10.3389/fbioe.2023.1254661 (PMC10507691; doi:10.3389/fbioe.2023.1254661)
Supplement: Supplementary file 1 [file DataSheet1.PDF]

Indirect validation: EMG vs Muscle Activations

To indirectly validate the modeling outputs of KCFs, a quantitative comparison between the processed EMG data and the corresponding muscle activations predicted by the models were performed. The EMG peaks were normalized to the corresponding activation peaks. Specifically, Pearson correlation coefficients (R) and root-mean-squared errors (RMSE) between the 2 variables averaged over the stance phase of the gait cycles for the three motor activities were calculated and reported in the following Table S1. Figures S1, S2 and S3 show the comparison across the motor activity cycles.

The comparison between predicted muscle activations and measured EMGs showed moderate to marked significant correlations ($0.58 < R < 0.91$ during walking, $0.32 < R < 0.98$ during stair ascending, $0.56 < R < 0.89$ during stair descending) and low RMSE (RMSE < 0.185 during walking, RMSE < 0.091 during stair ascending, RMSE < 0.108 during stair descending) in all motor activities. The few exceptions included Rectus Femoris during walking and stair ascending, which showed inverse correlation, and Medial Gastrocnemius and Tibialis Anterior during stair descending, which showed no significant correlation and inverse correlation respectively.

Table S1. Pearson correlation coefficients (R) and root-mean-squared errors (RMSE) averaged over the stance phase of the gait cycles for each motor activity

**Muscles**	**Walking**			**Stair Ascending**			**Stair Descending**		
	R	p-value	RMSE	R	p-value	RMSE	R	p-value	RMSE
Gluteus Medius	0.58	0.000	0.185	0.94	0.000	0.074	0.82	0.000	0.108
Erector Spinae	0.69	0.000	0.047	0.86	0.000	0.045	0.56	0.000	0.041
Rectus Femoris	-0.43	0.000	0.109	-0.21	0.032	0.065	0.72	0.000	0.061
Vastus Medialis	0.87	0.000	0.023	0.98	0.000	0.053	0.89	0.000	0.061
Biceps Femoris LH	0.89	0.000	0.038	0.70	0.000	0.091	0.63	0.000	0.026
Semitendinosus	0.91	0.000	0.017	0.32	0.001	0.014	0.56	0.000	0.014
Gastrocnemius MH	0.91	0.000	0.148	0.97	0.000	0.056	-0.08	0.442	0.146
Tibialis Anterior	0.79	0.000	0.058	0.38	0.000	0.020	-0.72	0.000	0.023

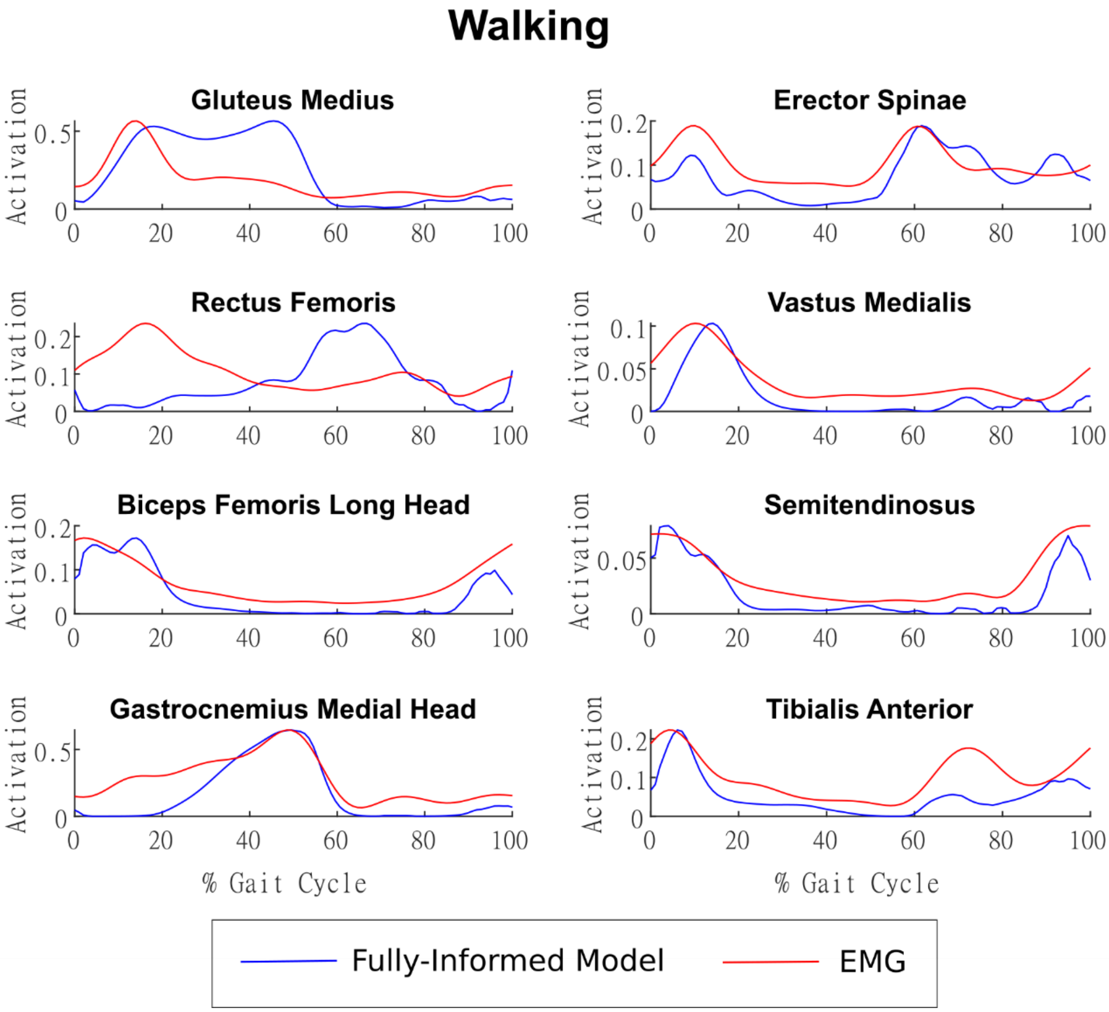

Figure S1. Comparison of predicted muscle activations and measured during walking averaged over all the patients. EMG peaks are normalized to the corresponding activation peaks.

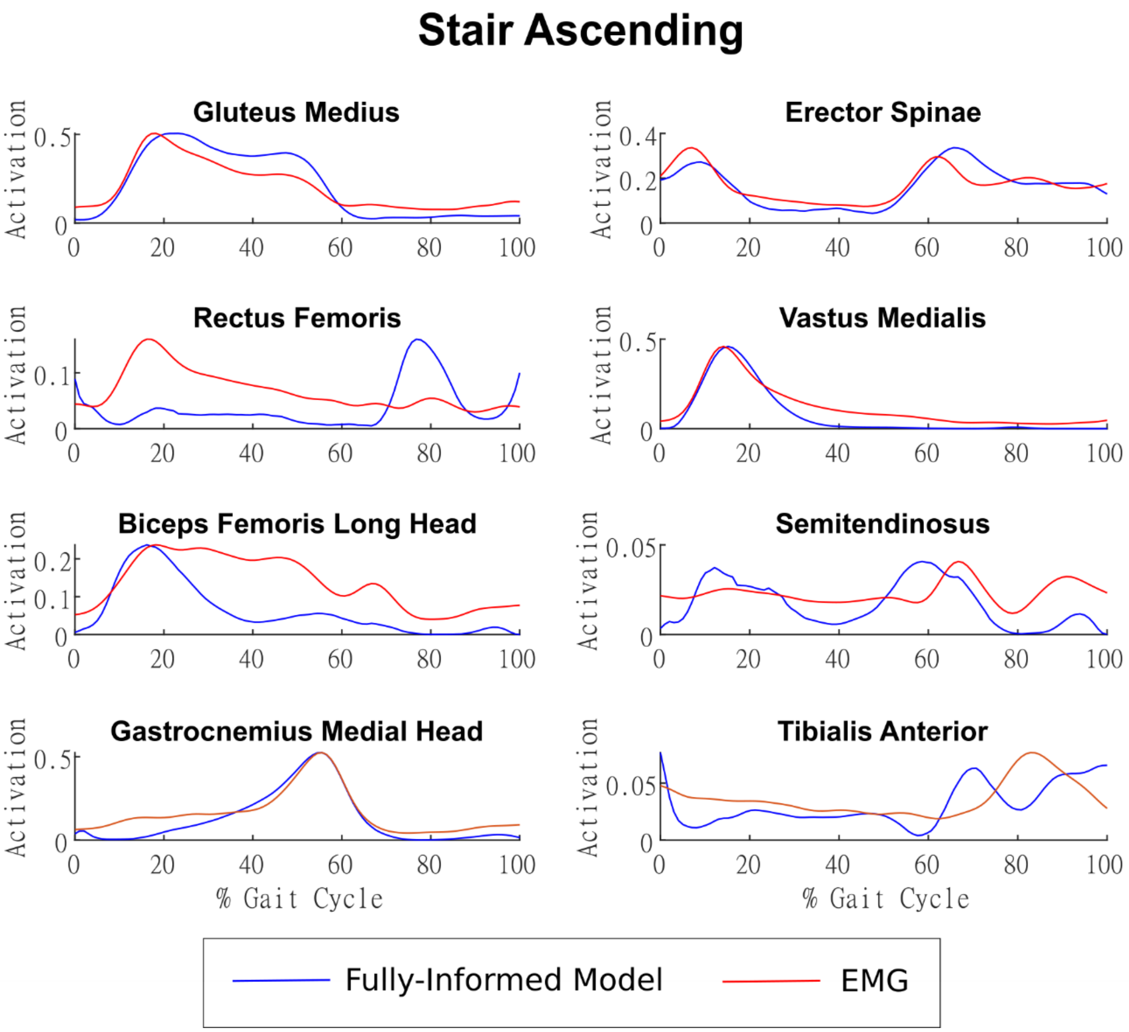

Figure S2. Comparison of predicted muscle activations and measured during stair ascending averaged over all the patients. EMG peaks are normalized to the corresponding activation peaks.

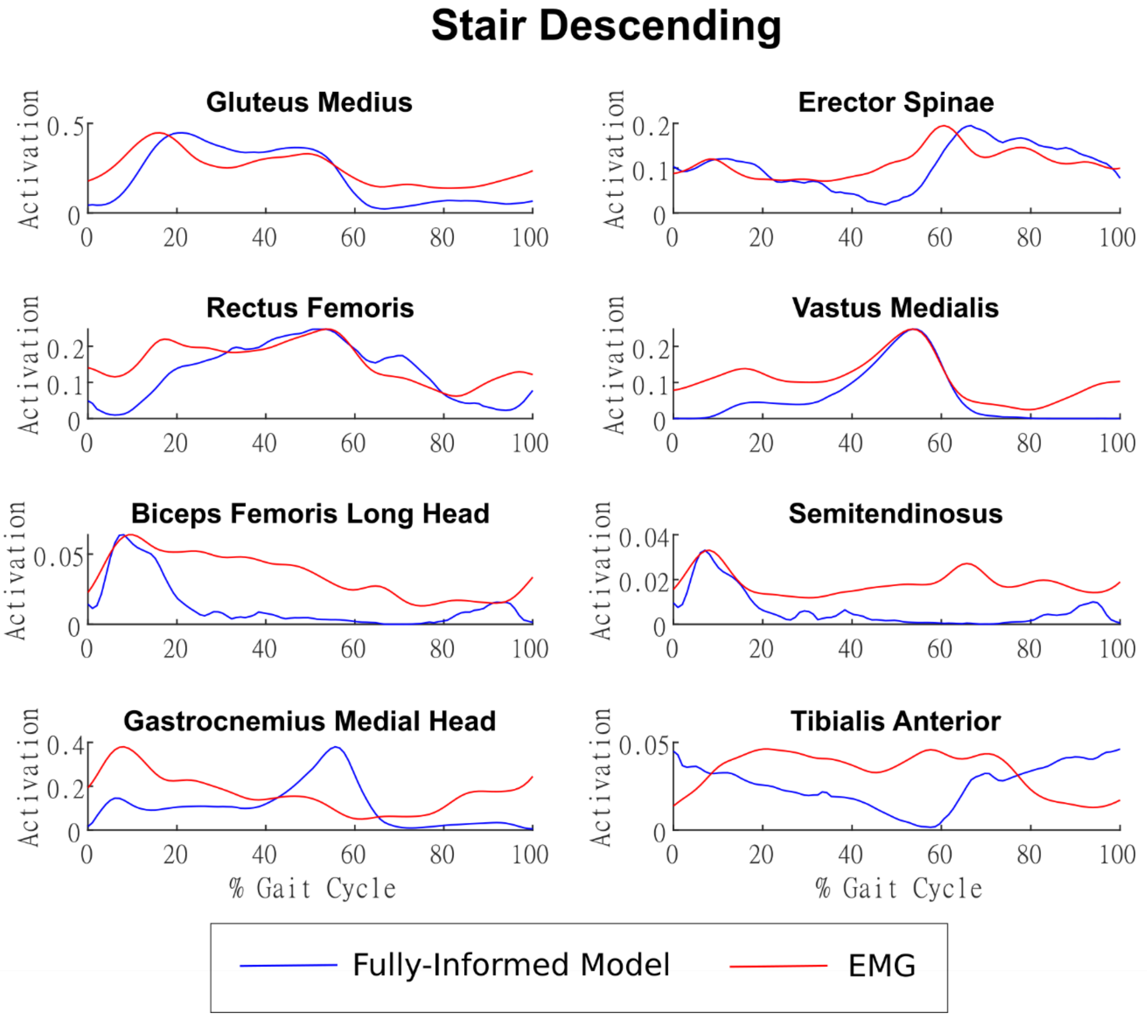

Figure S3. Comparison of predicted muscle activations and measured during stair descending averaged over all the patients. EMG peaks are normalized to the corresponding activation peaks.
